# Animal Ecosystem Engineers Modulate the Diversity-Invasibility Relationship

**DOI:** 10.1371/journal.pone.0003489

**Published:** 2008-10-22

**Authors:** Nico Eisenhauer, Alexandru Milcu, Alexander C. W. Sabais, Stefan Scheu

**Affiliations:** 1 Darmstadt University of Technology, Institute of Zoology, Darmstadt, Germany; 2 NERC Centre for Population Biology, Division of Biology, Imperial College London, Silwood Park Campus, Ascot, Berkshire, United Kingdom; Centre National de la Recherche Scientifique, France

## Abstract

**Background:**

Invasions of natural communities by non-indigenous species are currently rated as one of the most important global-scale threats to biodiversity. Biodiversity itself is known to reduce invasions and increase stability. Disturbances by ecosystem engineers affect the distribution, establishment, and abundance of species but this has been ignored in studies on diversity-invasibility relationships.

**Methodology/Principal Findings:**

We determined natural plant invasion into 46 plots varying in the number of plant species (1, 4, and 16) and plant functional groups (1, 2, 3, and 4) for three years beginning two years after the establishment of the Jena Experiment. We sampled subplots where earthworms were artificially added and others where earthworm abundance was reduced. We also performed a seed-dummy experiment to investigate the role of earthworms as secondary seed dispersers along a plant diversity gradient. Horizontal dispersal and burial of seed dummies were significantly reduced in subplots where earthworms were reduced in abundance. Seed dispersal by earthworms decreased with increasing plant species richness and presence of grasses but increased in presence of small herbs. These results suggest that dense vegetation inhibits the surface activity of earthworms. Further, there was a positive relationship between the number of earthworms and the number and diversity of invasive plants. Hence, earthworms decreased the stability of grassland communities against plant invasion.

**Conclusions/Significance:**

Invasibility decreased and stability increased with increasing plant diversity and, most remarkably, earthworms modulated the diversity-invasibility relationship. While the impacts of earthworms were unimportant in low diverse (low earthworm densities) and high diverse (high floral structural complexity) plant communities, earthworms decreased the stability of intermediate diverse plant communities against plant invasion. Overall, the results document that fundamental processes in plant communities like plant seed burial and invader establishment are modulated by soil fauna calling for closer cooperation between soil animal and plant ecologists.

## Introduction

Invasions of natural communities by non-indigenous species are currently rated as one of the most important global-scale environmental problems [1], [2]. The loss of biodiversity has generated concern over the consequences for ecosystem functioning and thus understanding the relationship between both has become a major focus in ecological research during the last two decades [3]–[6]. The “biodiversity-invasibility hypothesis” by Elton [7] postulates that high diversity increases the competitive environment of communities and makes them more difficult to invade. Numerous biodiversity experiments have been conducted since Elton's time and several mechanisms have been proposed to explain the often observed negative relationship between diversity and invasibility. Beside the decreased chance of empty ecological niches but the increased probability of competitors that preclude invasion success, diverse communities are assumed to use resources more completely and, therefore, limit the ability of invaders to establish [5], [7], [8]. Further, more diverse communities are believed to be more stable because they use a broader range of niches than species-poor communities [5], [7]–[10].

Plant community composition results from dynamics in plant mortality and seedling establishment. Thereby, local processes like small scale disturbances are essential factors driving the establishment of seedlings [11], [12]. In this context, ecosystem engineering, i.e. the modification, maintenance, creation or destruction of habitats, clearly has the potential to affect the distribution, establishment and abundance of species [13], [14]; e.g. ants considerably alter the vegetation structure in grassland by creating gaps and dispersing plant seeds [15]. Surprisingly, however, ecosystem engineers have been ignored in studies investigating the diversity-invasibility relationship. Thus, considering ecosystem engineers is essential for a more complete understanding of the factors driving the invasibility and stability of plant communities.

It is increasingly recognized that after primary seed dispersal, i.e. the displacement of seeds form the parent to the soil surface, large earthworm species feeding at the soil surface (anecic earthworm species), such as *Lumbricus terrestris* L. (Lumbricidae), play an important role in secondary seed dispersal, i.e. the subsequent dispersal of seeds on the soil surface and burial into the soil [12], [16]–[19]. Selective ingestion and digestion of seeds [20]–[22], horizontal and vertical (downward or upward) seed transport [16], acceleration [22], [23] or delaying of seed germination [16], [24] are the main mechanisms by which earthworms affect seedling establishment, and these processes likely are important for seedling mortality and establishment under natural conditions [19], [25]. Plant seed survival is primarily driven by processes during secondary seed dispersal, including horizontal and vertical movements (burial) and post-dispersal seed predation [26]. One of the most important and widely studied influences on seed survival is post-dispersal seed predation altering the number and distribution of seeds [27]. In some perennial communities, aboveground seed predation may destroy more than 95% of the seeds produced [28]. Seed burial by anecic earthworms therefore might be an essential mechanism to escape aboveground seed predation by vertebrates and ants [29]. However, since earthworm performance is driven by the plant community composition [30], earthworm impacts on the invasibility and stability of plant communities might depend on the diversity of plant communities. Earthworm effects might therefore be of minor importance in low diverse plant communities (due to low earthworm numbers) but likely are more important in diverse plant communities (due to high earthworm densities) [30].

We report results from three years of natural plant invasion into experimental grassland communities of the Jena Experiment [31]. To our knowledge the present study is the first focusing on the mechanisms of invasion susceptibility (invasibility) and stability (coefficient of variation in invader numbers and biomass) in a plant diversity gradient as modulated by ecosystem engineers. We specifically hypothesized that (1) earthworms are important agents in secondary plant seed dispersal and that (2) earthworms thereby affect the diversity-invasibility relationship and plant community stability by increasing the invasibility and decreasing the stability of grassland plant communities.

## Materials and Methods

### Experimental setup

The present study was part of the Jena Experiment [31]. The study site is located on the floodplain of the Saale river at the northern edge of Jena (Thuringia, Germany). Mean annual air temperature 3 km south of the field site is 9.3°C and annual precipitation is 587 mm [32]. The site had been used as an arable field for the last 40 years and the soil is an Eutric Fluvisol [33].

**Figure 1:**
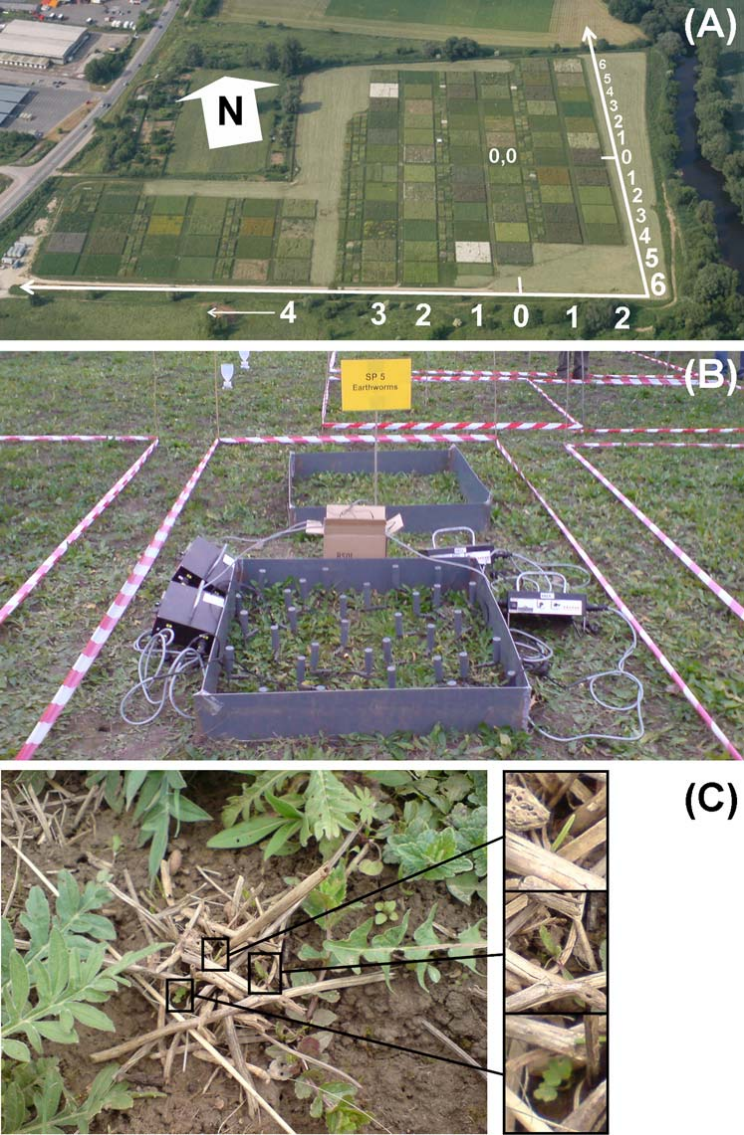
Experimental setup and earthworm midden. (A) Photograph of the field site of the Jena Experiment taken in 2004 showing the main experimental plots (20×20 m) varying in plant species richness (1, 2, 4, 8, 16, and 60) and plant functional group richness (1, 2, 3, and 4) and the X- (horizontal axis; coordinates 0–4) and Y-coordinates (vertical axis; coordinates 0–6). Photo by J. Baade. (B) Photograph of one exemplary earthworm subplot (1×1 m), the enclosures for earthworm density manipulations (earthworm addition and earthworm reduction), and four octet devices used for earthworm extraction by electro-shocking. Photo by N. Eisenhauer. (C) Photograph of one exemplary *Lumbricus terrestris* midden with three invader seedlings on the field site of the Jena Experiment. Photo by N. Eisenhauer.

The experiment was established in May 2002. The studied system represents Central European mesophilic grassland traditionally used as hay meadow (*Arrhenatherion* community). A pool of 60 native plant species was used to establish a gradient of plant species richness (1, 2, 4, 8, 16, and 60) by independent random draws with replacement. This represented a total of 82 plots of 20×20 m (Fig. 1A) [31]. Plant species were aggregated into four plant functional groups: grasses (16 species), small herbs (12 species), tall herbs (20 species), and legumes (12 species) by using (1) above- and belowground morphological traits, (2) phenological traits, and (3) the ability for N_2_ fixation as attribute classes [31]. The plots also encompassed a gradient in functional group richness (1, 2, 3, and 4) in a factorial design. Experimental plots were mown twice a year (June and September), as is typical for hey meadows, and weeded twice a year (April and July) to maintain the target species composition.

Plots were assembled into four blocks following a gradient in soil characteristics; each block contained an equal number of plots and plant species and functional group richness levels. Plots were divided into subplots to allow for the establishment of nested project-specific treatments and destructive measurements. Further information on the design and setup of the Jena Experiment is given in Roscher et al. [31].

### Manipulation of earthworm densities

Subplots where earthworm densities were manipulated were established on the 1 (16 replicates), 4 (16 replicates) and 16 plant species richness levels (14 replicates) in September 2003. In each plot, three randomly selected subplots of 1×1 m were used to establish the following treatments: control, earthworm addition and earthworm reduction. Subplots were enclosed with PVC shields aboveground (20 cm) and belowground (15 cm) to prevent the escape or colonization of *L. terrestris* which is the only large surface active (anecic) earthworm species occurring at the field site of the Jena Experiment. Earthworm addition subplots (+ew) received 25 adult individuals of *L. terrestris* (average fresh weight with gut content 4.10±0.61 g) per year (15 individuals in spring and 10 in autumn). The earthworm addition treatment was established since earthworm density was low after establishment of the Jena Experiment which involved repeated disk cultivation to reduce weed density, a practice which is known to detrimentally affect earthworms [34]. Further, two earthworm extraction campaigns were performed per year (spring and autumn) on the adjacent earthworm reduction subplots (-ew) by electro-shocking (Fig. 1B). A combination of four octet devices (DEKA 4000, Deka Gerätebau, Marsberg, Germany) [35] was used. In each subplot earthworm extraction was performed for 35 minutes, increasing the voltage from 250 V (10 min) to 300 V (5 min), 400 V (5 min), 500 V (5 min), and 600 V (10 min). Extracted earthworms were identified, counted and weighed in the laboratory (not shown).

### Seed dummy experiment

A seed dummy experiment was performed in May 2006, five weeks after the last earthworm density manipulation, to investigate the efficiency of density manipulations for *L. terrestris* (via earthworm soil surface activity) and to test our hypothesis (1) suggesting that anecic earthworms are important agents in secondary plant seed dispersal. Since *L. terrestris* is known to bury seeds irrespective of size and shape [12], [19], nine seed dummies (little glass beads; diameter 2 mm) spaced 25 cm were deployed in each earthworm treatment (control, earthworm addition, and earthworm reduction). Each seed dummy was marked with a flag to allow detecting the movement and burial of the dummies. The number of moved and buried dummies was determined one week after application. There was no heavy rain and wind during the experiment which could have moved the dummies. To evaluate potential impacts of voles, the number of vole holes was determined per subplot; it was not correlated with the number of moved and buried seed dummies (not shown). Thus, any movement of seed dummies was ascribed to earthworm activity.

### Plant invaders

All plant individuals which did not belong to the respective initial target plant community were considered invaders and were weeded from earthworm subplots in three consecutive years (April 2004, 2005, and 2006) to investigate if earthworms affect the diversity-invasibility relationship [hypothesis (2)]. Focusing on the main mechanisms of plant invader establishment, we did not distinguish between invaders belonging to the experimental species pool and non-experimental invader species. Weeded plants were identified, counted, separated into plant functional groups (grasses, herbs and legumes), dried (60°C, 72 h) and weighed. Invader diversity was determined by counting only herb and legume invader species per subplot; grass invaders (mostly as seedlings) were not considered since they were not identified to species level.

Further, we determined the stability of the plant communities by calculating the variability in plant invasion resistance as affected by plant diversity and earthworms [hypothesis (2)]. The coefficient of variation (CV; [%]) of the number and biomass of grass and herb invaders at the three weeding dates was used as measure of variability:
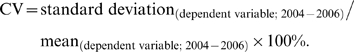



### Statistical analysis

All datasets were tested for normal distribution and homogeneity of variance and log-transformed (log_10_[x+1]), if necessary. Split plot ANOVA (GLM, type I sum of squares) was used to analyze the effects of block (B), plant species richness (S), plant functional group richness (Fg), and presence/absence of grasses (Gr), small herbs (Sh), tall herbs (Th), and legumes (Leg), and earthworms (Ew; control, earthworm addition, earthworm reduction) on the number of moved and buried seed dummies in a hierarchical order. Further, split plot ANCOVA (GLM, type I sum of squares) was used to analyze the effects of time (Ti), x- and y-coordinates (X and Y), S, Fg, Gr, Sh, Th, Leg, Ew (earthworm addition and earthworm reduction) and the interactions of time with the other factors on the number and biomass of total plant invaders, grass invaders, herb invaders, and plant invader diversity in April 2004, 2005, and 2006. Data on legume invaders were not analyzed separately and were not considered because of low numbers. X and Y were fitted as covariates to account for possible edge effects of seed import to experimental plots (X for east-west direction and Y for north-south direction; Fig. 1A). Earthworm treatments resembled “subplots” and sampling times resembled “sub-subplots” in the split plot ANCOVA [36]. To investigate effects of time and interactions of time with other factors orthogonal contrasts in repeated measures ANOVA were calculated [37]. In this approach polynomial contrasts are formed and tested against their own error terms [38], [39]. This avoids the problem of serial correlation and, therefore, the need to adjust the degrees of freedom [38], [39]. Since we analyzed differences between three years (2004, 2005, and 2006) only linear (Ti_linear_) and quadratic (Ti_quadratic_) contrasts were tested. However, for a detailed analysis of the single years separate protected split plot ANCOVAs were calculated [36], i.e. that the significance of the factor time in the overall analysis allowed for single analyses of the respective years. Moreover, split plot ANCOVA (GLM, type I sum of squares) was used to analyze the effects of X, Y, S, Fg, Gr, Sh, Th, Leg, and Ew on the CV of the number and biomass of grass and herb invaders.

F-values given in the text refer to those where the respective factor (and interaction) was fitted first [40]. B or X and Y were always fitted first, followed by S and Fg. Then, the effects of presence/absence of certain plant functional groups were calculated followed by Plot, Ew, and interactions between Ew and S and Fg, respectively. As stated above, for seed dummy movement and burial the factor B was used instead of X and Y since variations in abiotic soil parameters were more important than distance to the edge of the field site. Treatments analyzed at the plot scale (B, S, Fg, Gr, Sh, Th, and Leg) were tested against the variance between plots to avoid pseudoreplication, whereas treatments analyzed on the subplot scale (Ew, Ew×S, and Ew×Fg) were tested against the variance between subplots [36].

After fitting the full model, a minimum adequate model was derived using the Akaike Information Criterion (AIC) [41]. Analyses of variance and comparisons of means (Tukey's HSD test, α<0.05) were performed using SAS V9.1 (SAS Institute Inc., Cary, USA). Means (±S.E.) presented in text and figures were calculated using non-transformed data.

## Results

### Seed dummy experiment

**Figure 2:**
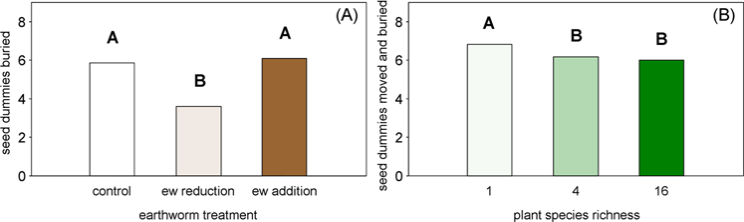
Seed dummies. (A) Effects of earthworm treatment (control, earthworm [ew] reduction, and earthworm addition) on the number of buried seed dummies after seven days (May 2006) and (B) effects of sown plant species richness (1, 4, and 16-species mixtures) on the number of moved and buried seed dummies after seven days (May 2006). Means with different letters vary significantly (Tukey's HSD test, α<0.05).

On average 6.32±0.14 seed dummies were moved and buried one week after the start of the experiment of which 4.04±0.16 seed dummies were buried. The number of moved and buried seed dummies differed strongly between earthworm treatments. They were similar in the control (6.76±0.12) and earthworm addition treatment (7.00±0.12), but considerably lower in the earthworm reduction treatment (5.20±0.12; F_2,80_ = 33.05, P<0.001). The number of buried seed dummies was even more reduced in earthworm reduction treatments (2.80±0.12) compared to control (−38%; 4.57±0.15) and earthworm addition treatments (−41%; 4.74±0.15; F_2,80_ = 36.94, P<0.001, Fig. 2A) indicating that earthworm soil surface activity was strongly reduced. Moreover, the number of buried seed dummies was higher in mixtures with three and four than in those with one and two plant functional groups in the control treatment. On the contrary, in earthworm addition and reduction treatments the highest numbers of seed dummies were buried in plant functional group monocultures (F_(Ew×S) 6,80_ = 3.68, P = 0.003).

Significantly more seed dummies were moved and buried in monocultures (6.79±0.13) than in 4-species (6.15±0.13) and 16-species mixtures (5.98±0.15, F_2,34_ = 3.26, P = 0.049; Fig. 2B), however, the number of buried seed dummies was not affected by plant species richness (F_2,34_ = 1.99, P>0.1). Further, the presence of grasses reduced the number of moved and buried (−10%; F_1,34_ = 4.11, P = 0.047) and the number of buried seed dummies (−17%; F_1,34_ = 5.01, P = 0.032). On the contrary, the presence of small herbs increased the number of moved and buried seed dummies slightly but significantly (+3%; F_1,34_ = 5.30, P = 0.027) and the number of buried seed dummies in trend (+11%; F_1,34_ = 3.04, P = 0.075).

### Invader number and biomass

Impacts of soil abiotic characteristics (block or x- and y-coordinates), plant diversity, and presence of certain plant functional groups on the invasibility of the plant community are not topic of the present study but are illustrated in brief to provide background information necessary to interpret results on invader success.

Generally, the number and biomass of plant invaders increased during the three years of experimental weeding from 51.1±6.8 and 21.5±4.1 g/m^2^ in 2004 to 63.1±11.2 and 32.7±5.1 g/m^2^ in 2005 and 92.4±10.3 and 61.8±10.6 g/m^2^ in 2006 (F_(linear) 1,37_ = 20.89, P<0.001, F_(quadratic) 1,37_ = 2.87, P<0.1 and F_(linear) 1,37_ = 33.66, P<0.001, F_(quadratic) 1,37_ = 0.38, P>0.1, for number and biomass of plant invaders, respectively). Further, x-coordinate fitted as covariate generally did not significantly affect the number and biomass of plant invaders, however, the y-coordinate significantly affected the number of herb invaders in 2006 with lower numbers in the center than at the edge of the field site (not shown).

Plant species richness strongly affected the number and biomass of plant invaders (invaders total, invader grasses and invader herbs) at each of the three weeding dates. Generally, the number and biomass of plant invaders significantly decreased with increasing plant species and plant functional group richness (not shown). Further, total invader biomass (2004: −80%, 2005: −85% ), number (2004: −73%, 2005: −89%, 2006: −20%) and biomass (2004: −86%, 2005: −89%) of grass invaders, and biomass of herb invaders (2004: −73%) were significantly decreased in presence of grasses (not shown). By contrast, total invader biomass (2004: +17%), and number (2004: +15%) and biomass (2004: +48%, 2006: +132%) of grass invaders were increased in presence of legumes (not shown). Further, while the number of total invaders was increased in 2006 (+111%), grass invader biomass was slightly decreased in presence of tall herbs in 2005 (−3%; not shown).

Moreover, the number of grass invaders decreased in trend in earthworm addition compared to reduction treatments in 2004 (−19%; F_1,40_ = 3.47, P = 0.07). By contrast, the total number of invader plants (+10%) and the number of invader grasses (+18%) were increased in trend in earthworm addition treatments compared to reduction treatments in 2006 (F_(total) 1,40_ = 3.04, P = 0.09 and F_(grasses) 1,40_ = 3.60, P = 0.06). However, there was no interactive impact of plant diversity and earthworm treatment on the invasibility of the plant community (not shown).

### Invader diversity

Invader diversity changed during the three years with lower numbers in 2005 (1.54±0.13 invader species) than in 2004 (3.15±0.26 invader species) and 2006 (2.83±0.14 invader species) (F_(linear) 1,128_ = 0.84, P>0.1, F_(quadratic) 1,128_ = 29.98, P<0.001).

Plant invader diversity decreased significantly from monocultures (4.22±0.26, 2.38±0.15, and 3.63±0.13 invader species in 2004, 2005 and 2006, respectively) to 4-species mixtures (3.44±0.25, 1.47±0.10, and 2.97±0.12 invader species) and 16-species mixtures (1.61±0.20, 0.68±0.08, and 1.75±0.07 invader species; F_(2004) 2,34_ = 5.76, P = 0.007; F_(2005) 2,34_ = 12.55, P<0.001; F_(2006) 2,34_ = 13.88, P<0.001). Moreover, increasing plant functional group richness decreased plant invader diversity significantly in 2005 from 2.23±0.13 invader species in single plant functional group treatments to 1.31±0.13, 0.63±0.08, and 0.81±0.06 invader species in mixtures with two, three and four plant functional groups, respectively (F_3,34_ = 8.70, P<0.001). Similarly, plant invader diversity also decreased with increasing plant functional group richness in 2006 from 3.41±0.14 invader species in single plant functional group treatments to 2.56±0.11, 2.19±0.13, and 2.12±0.10 invader species in mixtures with two, three and four plant functional groups, respectively (F_3,34_ = 4.78, P = 0.007). Presence/absence of certain plant functional groups affected plant invader diversity only in 2006 with a decrease in invader diversity in presence of tall herbs (−5%; F_1,34_ = 5.95, P = 0.02) and legumes (−34%; F_1,34_ = 5.96, P = 0.02). Further, plant invader diversity was significantly increased in earthworm addition treatments compared to reduction treatments (+12%; F_1,40_ = 4.40, P<0.05). However, this did not depend on plant species richness (F_2,40_ = 0.61, P>0.1) and plant functional group richness (F_3,40_ = 0.73, P>0.1).

### Stability

The variability of the number and biomass of grass (98% and 106%, respectively) and herb invaders (83% and 97%, respectively) was high. Fitting the x- and y-coordinates as covariates suggests that the CV of grass and herb invaders did not depend on the distance from the edge of the experimental field site (number and biomass; not shown). Further, plant functional group richness, and the presence of small herbs, tall herbs, and legumes did not affect the CV of the number and biomass of grass invaders. However, the CV was lower in 16-species mixtures (64 and 61% for invader number and biomass, respectively) than in monocultures (97 and 117%) and in 4-species mixtures (128 and 135%; F_(number) 2,34_ = 5.50, P = 0.01 and F_(biomass) 2,34_ = 15.15, P<0.001). Presence of grasses did not affect the CV of the number of invader grasses but decreased the CV of the biomass of invader grasses considerably (−35%; F_1,34_ = 13.68, P<0.001). Moreover, the CV of the number of grass invaders was increased in trend in the earthworm addition treatment (+11%; F_1,40_ = 3.52, P = 0.07).

**Figure 3:**
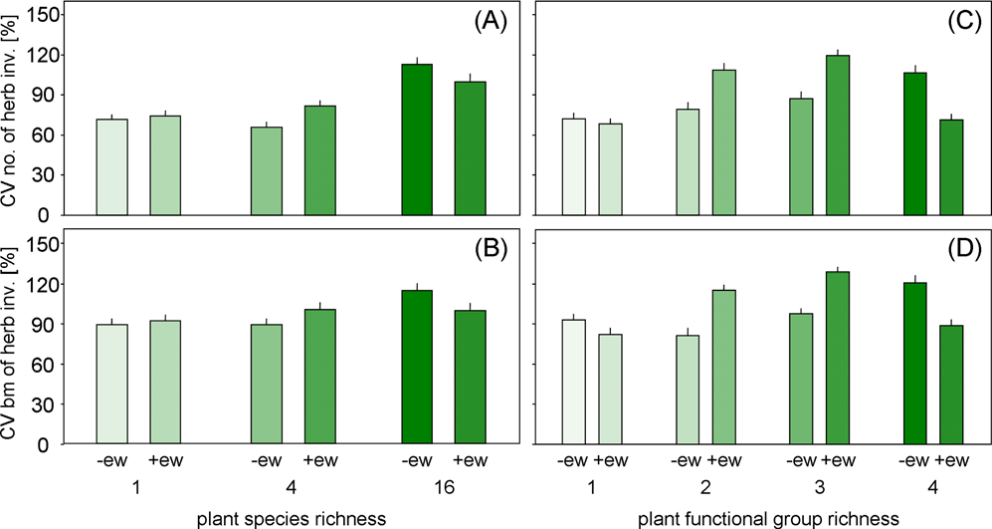
Interacting effects of plant diversity and earthworms. Effects of plant species richness (1, 4, and 16-species mixtures) and earthworm treatment (earthworm reduction [-ew] and earthworm addition [+ew]) on the coefficient of variation (CV; [%]) of (A) the number and (B) biomass of herb invaders in the years 2004 to 2006 and effects of plant functional group richness (1, 2, 3, and 4 plant functional groups) and earthworm treatment on the CV [%] on (C) the number and (B) biomass of herb invaders in the years 2004 to 2006. Means with standard errors.

Although the CV of the number and biomass of herb invaders was not affected by plant diversity and the presence of certain plant functional groups (not shown), the interactions between earthworm treatment and plant species and functional group richness had significant effects (Fig. 3). While the CV of the number and biomass of herb invaders did not differ in monocultures, the respective CVs were increased in earthworm addition treatments in 4-species mixtures (+25% and +13% for invader number and biomass, respectively) but decreased in 16-species mixtures (−11% and −13%; F_(number) 2,40_ = 3.50, P = 0.04 and F_(biomass) 2,40_ = 4.66, P = 0.015; Fig. 3A, B). Similarly, the CV of the number and biomass of herb invaders did not differ in single plant functional group treatments, however, they were increased in earthworm addition treatments in mixtures with two (+37% and +42% for invader number and biomass, respectively) and three plant functional groups (+37% and +32%) but decreased in mixtures with four plant functional groups (−33% and −26%; F_(number) 3,40_ = 3.18, P = 0.034 and F_(biomass) 3,40_ = 4.33, P = 0.01; Fig. 3C, D).

## Discussion

### Secondary seed dispersal

Long-term density manipulations of soil invertebrates in the field are labor-intensive and may not be perfectly successful. By adding earthworms to our field site we intended to increase the densities of *L. terrestris* since they were low after the establishment of the Jena Experiment (A. Milcu, unpubl. data). However, four years after establishment earthworm densities were saturated as indicated by similar earthworm soil surface activities (seed-dummy experiment) in earthworm addition and control treatments. On the contrary, earthworm soil surface activity was decreased significantly by −38% in earthworm reduction plots. Considering that our manipulations only reduced the impact of earthworms on seed dispersal and burial, the experiment only reflects part of the full effects of earthworms on plant communities and invader success. Indeed, greenhouse experiments suggest that earthworms strongly affect the fate of plant seeds and seedling recruitment [12], [19]. By successfully manipulating earthworm densities in the field the present study for the first time documents that earthworms in fact function as essential agents in secondary plant seed dispersal in grassland ecosystems confirming our hypothesis (1).

### Invasibility

During the first two years of the experiment the effects of earthworms on plant invader establishment were non-significant. However, in the third year after establishment there was a distinct trend of increased numbers of total and grass invaders and a significant increase in plant invader diversity in earthworm addition plots. Results of the seed-dummy experiment indicate that earthworms likely modulated plant invasion and invader establishment success by dispersal and burial of plant seeds. Indeed, greenhouse experiments showed that earthworms disperse, bury, swallow and digest plant seeds and thereby alter plant community assembly [12], [19], [22], [42]. Interestingly, earthworm–plant seed interactions vary with plant species and are driven by seed size, shape and surface structure [42]. Moreover, there is field evidence that seed predation and transport are important mechanisms by which earthworms can alter the diversity of grassland ecosystems [25]. Remarkably, Grant [16] found 70% of the seedlings in temperate grasslands to germinate from earthworm casts, although casts only covered about 25% of the soil surface. Earthworm middens and casts represent nutrient-rich patches with comparatively low competition with the resident plant community [12] which likely facilitates seedling establishment (Figure 1C) thereby compensating seed loss due to digestion. This indicates that earthworm middens presumably increase the spatial heterogeneity of grassland plant communities [19], [43]. Moreover, earthworm gut passage of grassland plant seeds was shown to mostly increase germination rates but to be earthworm- and plant species-specific [22], [42]. Further, Zaller and Arnone [44] reported distinct associations between earthworm casts and certain plant species in calcareous grassland. Generally, the establishment of seedlings depends strongly on local processes like small scale disturbances [11]. Though, earthworm middens likely represent small scale disturbances of intermediate strength which are known to increase diversity [45], [46].

### Stability

Milcu et al. [12] suggested that earthworms might increase the resilience of grassland communities by moving plant seeds from the seed bank to soil surface and Eisenhauer and Scheu [19] concluded from results of a greenhouse experiment that earthworms contribute to the positive relationship between plant species diversity and resistance against invaders. In contrast to these assumptions, our results indicate that at least in grassland communities of intermediate diversity rather the opposite is true. As described above, earthworm middens formed by anecic species represent small-scale disturbances increasing the invasibility and, thereby, decreasing the stability of grassland communities. Interestingly, this phenomenon depended on the diversity of the resident plant community with no effects in monocultures and high diverse plant communities (plots containing 16 species, or four plant functional groups).

As indicated by earthworm extractions on the field site of the Jena Experiment [30], monocultures maintained only low numbers and biomass of anecic earthworms. Consequently, earthworm effects on the plant community were of minor importance. Moreover, plots containing only one plant species or one plant functional group provided ample gaps for invader establishment (as indicated by the coverage of the plant community; not shown) attenuating possible earthworm effects. But why were earthworm effects missing in high diverse plant communities where earthworm biomass was high? The seed-dummy experiment showed that despite the high earthworm biomass and density [30], soil surface activity decreased with increasing plant species richness suggesting that the more dense vegetation [47] hampered finding and dispersal of plant seeds by *L. terrestris*. Consequently, in addition to reducing the number of open gaps, light and nutrient availability, diverse plant communities might be more stable against plant invasion due to less efficient soil surface activity of anecic earthworms. This conclusion is supported by the fact that earthworm soil surface activity was decreased and invasion resistance was increased in presence of grasses. Grasses produce large numbers of shoots thereby increasing the structural complexity of grassland plant communities. The associated reduction in earthworm surface activity likely contributed to the reduced numbers and biomass of *L. terrestris* in presence of grasses, and this further reduced invasibility. On the contrary, earthworm performance was increased in presence of legumes [30] and this probably contributed to the high invasibility of legume plant communities. Also, high earthworm surface activity in communities with small herbs, i.e. in plant communities with low structural complexity, likely contributed to the sensitivity of these communities to invaders. The present study indicates that plant species invasion and community stability is driven by a complex interaction between the diversity, functional identity and structural complexity of grassland plant communities, but also by belowground ecosystem engineers such as anecic earthworms. Thus, anecic earthworms indeed modulated the diversity-invasibility relationship and stability of grassland plant communities confirming our hypothesis (2). In contrast to our expectations, however, earthworm impacts were of minor importance in high diverse plant communities, likely due to high plant structural complexity.

### Conclusions

Generally, plant diversity and competition for resources are key factors driving the invasibility and stability of grassland communities. However, for comprehensively understanding species interactions and ecosystem functioning non-trophic interactions, such as ecosystem engineering, need to be considered. The present study highlights the intimate relationship between earthworms and plant diversity, functional group identity and structural complexity in the invasibility and stability of grassland communities. Fundamental processes in plant communities like secondary plant seed dispersal, plant community invasibility and stability were modulated by soil fauna components calling for closer cooperation of plant and animal ecologists.
